# The Role of Vasoactive Intestinal Peptide and Mast Cells in the Regulatory Effect of *Lactobacillus casei* ATCC 393 on Intestinal Mucosal Immune Barrier

**DOI:** 10.3389/fimmu.2021.723173

**Published:** 2021-11-25

**Authors:** Xiaofan Song, Shanyao Pi, Yueming Gao, Fengxia Zhou, Shuqi Yan, Yue Chen, Lei Qiao, Xina Dou, Dongyan Shao, Chunlan Xu

**Affiliations:** The Key Laboratory for Space Bioscience and Biotechnology, School of Life Sciences, Northwestern Polytechnical University, Xi’an, China

**Keywords:** probiotic, *Lactobacillus casei* ATCC 393, mast cells, vasoactive intestinal peptide, mucosal immune, intestinal barrier

## Abstract

Vasoactive intestinal peptide (VIP) plays an important role in the neuro-endocrine-immune system. Mast cells (MCs) are important immune effector cells. This study was conducted to investigate the protective effect of *L. casei* ATCC 393 on Enterotoxigenic *Escherichia coli* (ETEC) K88-induced intestinal mucosal immune barrier injury and its association with VIP/MC signaling by *in vitro* experiments in cultures of porcine mucosal mast cells (PMMCs) and *in vivo* experiments using VIP receptor antagonist (aVIP) drug. The results showed that compared with the ETEC K88 and lipopolysaccharides (LPS)-induced model groups, VIP pretreatment significantly inhibited the activation of MCs and the release of β-hexosaminidase (β-hex), histamine and tryptase. Pretreatment with aVIP abolished the protective effect of *L. casei* ATCC 393 on ETEC K88-induced intestinal mucosal immune barrier dysfunction in C57BL/6 mice. Also, with the blocking of VIP signal transduction, the ETEC K88 infection increased serum inflammatory cytokines, and the numbers of degranulated MCs in ileum, which were decreased by administration of *L. casei* ATCC 393. In addition, VIP mediated the regulatory effect of *L. casei* ATCC 393 on intestinal microbiota in mice. These findings suggested that VIP may mediate the protective effect of *L.casei* ATCC 393 on intestinal mucosal immune barrier dysfunction *via* MCs.

## Introduction

The intestinal barrier is essential for maintaining intestinal homeostasis and health. It prevents the loss of water and electrolytes and the invasion of antigens and microorganisms (1, 2). Therefore, the integrity of the intestinal barrier is particularly important for human and animal health. One of the main causes of gastrointestinal diseases such as necrotizing enterocolitis, irritable bowel syndrome (IBS) and inflammatory bowel disease (IBD) is the impairment of intestinal epithelial barrier integrity (3, 4). The intestinal mucosal immune barrier is mainly composed of intestinal mucosal epithelial cells and intestinal associated lymphoid tissues. Intestinal mucosal immune cells mainly include lymphocytes, goblet cells, mast cells, etc. The intestinal microbiome as a key component of the intestinal barrier system are strongly associated with the host health (5). *Lactobacillus casei* (*L. casei*) strains are commonly added to yogurt and fermented dairy products to improve their health benefits (6). *L. casei* could relieves constipation and diarrhea (7, 8). *L. casei* has also shown promise in preventing or alleviating human IBDs (9). However, the attenuation of colitis by *L. casei* BL23 was found to be dependent on the dairy delivery matrix (10). The impact of *L. casei* on the cecal microbiome and innate immune system is strain specific (11), and dose and time-dependent (12). *L. casei* expressing internalins A and B significantly reduced *Listeria monocytogenes*-induced cell cytotoxicity and epithelial barrier dysfunction (13). Moreover, *L. casei* ATCC 393 alleviated ETEC K88-induced intestinal barrier dysfunction *via* the toll-like receptors (TLRs)/mast cells (MCs) pathway (14).

MCs are an important natural immune effector cells, which participate in mucosal immunomodulatory process by releasing cytokines. The activation and degranulation of MCs is involved in the regulation of variety of physiological and pathological conditions in various settings (15). The reversal of ion permeation and transmembrane transport of macromolecules by MCs stabilizers was demonstrated in animal studies (16). MCs serve as a line of defense against antigens entering the body, and contribute to maintain the homeostasis of the immune system (17). MCs express various receptors including pathogen-associated molecular patterns (PAMPs), vasoactive intestinal peptide receptors (VPACs), NOD-like receptors (NLRs) as well as TLRs, all of which are involved in MC activation and immune response (18). A variety of endogenous and exogenous drugs can stimulate MCs to release mediators. The impairment of the intestinal barrier is related to the increase of intracavity antigens entering the mucosa, which further promotes the activation of MCs in the mucosa inflammatory response and changes in MC-enteric nerve interaction (19). Vasoactive intestinal peptide (VIP) plays a crucial role in the neuro-endocrine-immune system. Some studies have demonstrated the role of VIP in intestinal permeability regulation (20). Other studies have shown that MCs and VIP regulate the ileal barrier of healthy people and the stress response in rats through the VPAC1/VPAC2 receptors on the surface of MCs (21). An increase in plasma VIP levels was found in IBS patients and animal models. Also, intestinal epithelial permeability appears to be a positively correlated with mucosal MCs (22). However, the association of the protective effect of *L. casei* ATCC 393 on intestinal barrier function with VIP/MCs remains unclear.

This study was aimed to investigate the role of VIP in the protective effect of *L. casei* ATCC 393 on intestinal barrier dysfunction in mice challenged by ETEC K88 and its association with MCs. The ETEC K88 and LPS-induced VIP-mediated regulatory effect on MCs activation, and its association with the protective effect of *L. casei* ATCC 393 on intestinal epithelial barrier function were evaluated through *in vitro* co-culture experiments of porcine intestinal mucosal mast cells (PMMCs) with *L. casei* ATCC 393 and *in vivo* experiments using VIP receptor antagonist (aVIP) drug.

## Materials and Methods

### Bacterial Strains, Cell Line, and Reagents

*L. casei* ATCC 393 and ETEC K88 strain were kept in our laboratory. PMMCs were purchased from Saiqi (Shanghai) Biological Engineering Co., Ltd (Cat # CBR-131443). Man, Rogosa and Sharpe (MRS) broth (Cat # CM1153B) was purchased from Oxoid (Basingstoke, UK). Luria-Bertani (LB) broth (Cat # 12780052) were purchased from Gibco-Invitrogen. The reagents for cell culture were purchased from Invitrogen/Gibco (Carlsbad, CA, USA). Enzyme-linked immunosorbent assay (ELISA) Kits for porcine tumor necrosis factor-α (TNF-α, Cat# JL13203), porcine and mouse interferon-γ (IFN-γ, Cat# JL11792), porcine interleukin-6 (IL-6, Cat# JL21880), porcine interleukin-8 (IL-8, Cat#JL45446), porcine granulocyte-macrophage colony stimulating factor (GM-CSF, Cat#JL21931), porcine β-hexosaminidase (Cat# JL45717), porcine tryptase (Cat# JL17996), and porcine histamine (Cat#JL10076), and mouse TNF-α (Cat#JL10484), mouse IFN-γ (Cat#JL10967), mouse IL-6 (Cat#JL20268), mouse IL-1β (Cat#JL18442), mouse β-hexosaminidase (Cat#JL20214), mouse tryptase (Cat#JL20445), mouse histamine (Cat#JL10420), and mouse MPO (Cat#JL10367) were purchased from Jianglaibio Co., Ltd (Shanghai, China). The bicinchoninic acid (BCA) protein assay kit (Cat#P0012S) was purchased from Beyotime Biotechnology (Shanghai, China). VIP and VIP receptor antagonist (VIP_6-28_, aVIP) were synthesized by Shanghai Qiangyao Biological Co.,Ltd (Shanghai, China). Lipopolysaccharides (LPS, Cat#L4391) from *Escherichia coli* O111:B4 was purchased from Sigma Aldrich Company (Saint Louis, MO, USA). Primary antibodies for MUC2, Occludin, ZO-1, TLR4, NF-κB, p-NF-κB, MyD88 and β-actin were purchased from ABclonal Company (Wuhan, China).

### Cell Culture Conditions

PMMCs were cultured in high glucose Dulbecco’s modified Eagles’s medium (DMEM) supplemented with 10% fetal bovine serum (FBS) and 1% antibiotic mixture (100 U/mL of penicillin and 100 μg/mL streptomycin) in an incubator at 37°C in a humidified atmosphere with 5% CO_2_.

### Bacterial Culture Conditions

*L. casei* ATCC 393 was incubated in MRS broth at 37°C for 24 h without shaking. ETEC K88 was cultured in LB broth with shaking at a speed of 120 rpm at 37°C overnight. Bacteria pellets were collected by centrifuging at 5,000 × g at 4°C for 10 min, and then washed with phosphate-buffered saline (PBS). The obtained bacteria were suspended in FBS-free cell culture medium and diluted to different concentrations. The supernatants of 1×10^8^ CFU/mL *L. casei* ATCC 393 and 1×10^8^ CFU/mL of ETEC K88 culture medium were separately harvested by centrifugation at 5000 × g at 4°C for 10 min. The bacterial supernatants and bacterial resuspension solution were collected and used for subsequent experiments.

### Effect of VIP, aVIP and *L. casei* ATCC 393 on PMMCs Activation

PMMCs were seeded at a concentration of 4×10^5^ cells/mL in 24-well cell culture plates and cultured at 37°C for 24 h. Normal control cells were exposed to 1 mL of FBS-free DMEM. For the *L. casei* ATCC 393 treatment group, 1 mL of 1×10^8^ CFU/mL of *L. casei* ATCC 393 bacterial culture supernatants was added to each well. Other groups received 1 mL of FBS-free DMEM. VIP and aVIP treatment groups were administrated with 1 mL of FBS-free DMEM containing 0.1 μM VIP and/or 0.1 μM aVIP, respectively. The plates of all groups were incubated at 37°C for 12 h. After the above treatments, supernatants of cell culture medium supernatants were collected, and the concentration of β-hexosaminidase, tryptase, and histamine were determined using the corresponding ELISA kits according to the manufacture’s instruction.

### Effect of VIP and aVIP Interaction on the Activation of PMMCs

PMMCs were seeded at a concentration of 4×10^5^ cells/mL in 24-well cell culture plates and cultured at 37°C for 24 h. The cells were divided into four groups and treated accordingly: the normal control group received 1 mL of FBS-free DMEM; VIP and aVIP treatment group received 1 mL of FBS-free DMEM containing 0.1 μM VIP or/and 0.1 μM aVIP, respectively, and cultured at 37°C for 12 h. Afterwards, the cell culture medium supernatants were collected, and the concentration of β-hexosaminidase, tryptase, and histamine were determined using corresponding ELISA kits.

### Effect of *L.casei* ATCC 393 on VIP-Mediated Activation of PMMCs

PMMCs were inoculated at a density of 4×10^5^ cells/mL in sterile 24-well cell culture plate and cultured at 37°C for 24 h until the cell confluence reached higher than 90%. Normal control cells received 1 mL of FBS-free DMEM. The VIP alone group was treated with 1 mL of FBS-free DMEM containing 0.1 µM VIP for 12 h. The *L. casei* ATCC 393 alone group was exposed to 1 mL of 1×10^8^ CFU/mL *L. casei* ATCC 393 culture supernatant for 3 h. The VIP and *L. casei* ATCC 393 co-cultured cells were first administered with 1 mL of 1×10^8^ CFU/mL of *L. casei* ATCC 393 culture supernatant for 3 h, then changed to 1 mL of FBS-free DMEM containing 0.1 µM VIP and culured for 12 h. Afterwards, the cell culture supernatants were collected, and the concentration of β-hexosaminidase, tryptase and histamine were determined using the corresponding ELISA kits.

### Effect of VIP and aVIP on ETEC K88 or LPS-Induced PMMCs Activation

PMMCs were seeded at a density of 4×10^5^ cells/mL in sterile 24-well cell culture plates and cultured at 37°C for 24 h. First, normal control cells were exposed to 1 mL of FBS-free DMEM, the VIP alone treatment group was given 1 mL of FBS-free DMEM containing 0.1 µM VIP, and the aVIP treatment group received 1 mL of FBS-free DMEM containing 0.1 µM aVIP, and the aVIP-VIP co-treatment group received 1 mL of FBS-free DMEM containing 0.1 µM aVIP and 0.1 µM VIP. Then all group cells were cultured at 37°C for 12 h. Control groups were exposed to 1 mL of FBS-free DMEM, other experimental groups were changed to 1 mL of 1×10^8^ CFU/mL ETEC K88 culture supernatant or 1 mL of FBS-free DMEM containing 0.1 µM of LPS. After additional incubation for 2 h, cell culture medium supernatants were collected. The concentration of β-hexosaminidase, tryptase, histamine, IL-6, IL-8, TNF-γ and GM-CSF were determined by the corresponding ELISA kits.

### Animal Experimental Design

Animals can produce endogenous VIP, but whether it mediates the regulation of the *L. casei* ATCC 393 effect on intestinal barrier function remains unclear. We hypothesized that *L. casei* ATCC 393 can regulate the effect of pathogenic bacteria such as ETEC K88-induced intestinal mucosal MC activation and inhibit the release of MCs released mediators, regulate inflammatory responses, and further regulate intestinal barrier function through the binding of VIP and VIP receptors on the surface of intestinal mucosal MCs. To verify the above hypothesis, we conducted experiments to assess the effect of a VIP receptor antagonist (aVIP) in C57BL/6 mice challenged by ETEC K88. This animal experimental protocol was approved by the Laboratory Animal Welfare and Ethics Committee of Northwestern Polytechnical University and the experiment was conducted strictly in accordance with the International Laboratory Animal Assessment and Accreditation Committee guidelines for the care and use of laboratory animals. The 50 healthy male C57BL/6 mice (20 ± 2 g) used in the experiment were purchased from the Experimental Animal Center of Xi’an Jiaotong University (Xi’an, Shaanxi, China). The entire feeding experiment was conducted at the Experimental Animal Center of Northwestern Polytechnical University. After an adaptive period of 7 days, the mice were randomly divided into five groups with 10 mice per group: normal control group, ETEC K88 infected group, *L. casei* ATCC 393 protective group, aVIP + ETEC K88 treatment group, *L. casei* ATCC 393 + aVIP + ETEC K88 treatment group. The living conditions were as follows: relative humidity of 55 ± 5%, ambient temperature of 22 ± 5 °C, and under a 12 h light and dark cycle. The experimental scheme is depicted in **Figure 3A**. The mice in the *L. casei* ATCC 393 protective group were orally administered with 200 µL of 1×10^8^ CFU/mL of *L. casei* ATCC 393 resuspension solution per day for 14 days. The other groups were orally given the same volume of MRS broth. On days 1, 3, 5, 7, 9, and 11, mice in the ETEC K88-infected group were orally given 100 µL of 1×10^8^ CFU/mL ETEC K88 resuspension solution, and the other groups were given the same volume of LB broth. On days 0, 5 and 10, the aVIP treatment groups were intraperitoneally (i.p.) injected with aVIP (10 nmol/kg/BW). Other groups were i.p. injected with the same volume of normal saline. The body weight, diarrhea and mental status were observed and recorded daily. After the above treatments, mice were anesthetized with ether. Peripheral blood was drawn from mice and centrifuged immediately at 1,500×g for 15 min at 4°C to obtain serum. Serum samples were stored at -80°C until analyzed. Then, immediately the mice were dissected, the tissues of interest (the duodenum and ileum) were collected.

### Histological Analysis of Duodenum and Evaluation of Intestinal Barrier Function

The duodenum is an important intestinal segment of the digestive tract for nutrient digestion and absorption. Its histology and morphology are closely related to intestinal function. The tissues samples of the proximal duodenum were fixed in 10% neutral buffer formalin, dehydrate, and paraffin-embedded. Then, the sections prepared from the paraffin-embedded tissue blocks were stained with hematoxylin-eosin (H&E) and observed under a phase-contrast microscope for histological and morphological characterization of the duodenum. To further evaluate the changes in intestinal barrier function, the expression level of MUC2, Occludin, and ZO-1 proteins in duodenum were detected by immunofluorescence and Western Blot respectively. The expression levels of MUC2 and Reg3g genes were detected by q-PCR. Primer sequences are shown in **Table 1**.

**Table 1:** Primer sequences of MUC2 and Reg3g genes for q-PCR.

Gene product^a^	Primer	
	Direction^b^	Sequence (5’–3’)
MUC2	F	CAGACTACACGACAGGTGGG
	R	GTGGTGGTCGTTGATCCAGT
Reg3g	F	ATCAGCTGTCCCAAAGGCTC
	R	CATTTGGTTCCAAGCCCTCG

a MUC2, Mucin 2; Reg3g, Regenerating islet-derived protein III-gamma.

b F, forward; R, reverse.

### Detection of Intestinal Immune Responses

Intestinal mucosal MCs are key modulators of barrier function and homeostasis, which widely distributed in ileum. Therefore, the numbers of MCs and the degranulated MCs in proximal ileum were detected by toluidine blue (TB.) staining. Serum β-hexosaminidase, tryptase and MPO activities, as well as VIP, sIgA, histamine, TNF, IFN-γ, IL-6 and IL-1β concentrations were determined by the corresponding ELISA kits according to the manufacture’s instruction.

### Intestinal Microbiota Analysis

To investigate the changes of intestinal microbiota, we detected the cecal contents of mice by 16S rRNA amplicon sequencing. As in previous research methods, whole-genome DNA was extracted and sequenced for analysis (23). The sequences with similarity ≥97% were clustered. OTUs were assigned to each representative sequence in the cluster by searching against the GreenGene Database.

### Effects of aVIP and *L. casei* ATCC 393 on TLRs/MyD88/NF-κB Signaling Pathway

To further explore the mechanism of the effect on intestinal immune barrier function, we detected the protein expression levels of MyD88, TLR4, NF-κB (p65), and p-NF-κB (p-p65) by Western Blot.

### Statistical Analysis

All experimental data were statistically analyzed using Graphpad Prism 5.0 statistical software (GraphPad Software Inc., San Diego, CA, USA) and are presented as the mean ± standard error of mean (S.E.M.). The statistical significance was calculated by one-way analysis of variance (ANOVA) or Student’s *t*-test. Differences were considered significant at *P*<0.05. All assays were performed in at triplicate three independent experiments.

## Results

### Effect of VIP, aVIP and *L. casei* ATCC 393 on PMMCs Activation

**Figure 1:**
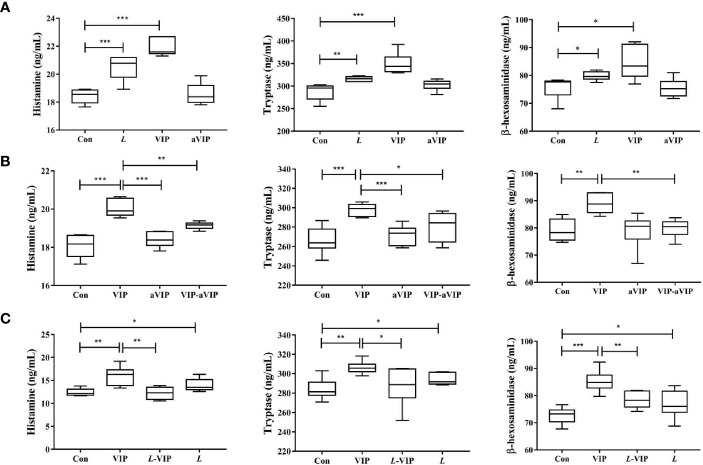
Effect of *L. casei* ATCC 393, vasoactive intestinal peptide (VIP), VIP receptor antagonist (aVIP), and the interaction between VIP and aVIP, *L. casei* ATCC 393 and VIP on the activation of porcine mucosal mast cells (PMMCs). The cells were treated with the supernatants of 1×10^8^ CFU/mL *L. casei* ATCC 393 culture medium for 12h. The cells were treated with 0.1μM VIP and aVIP in the same way. **(A)** The effect of *L. casei* ATCC 393, VIP, aVIP on the activity of β-hexosaminidase, tryptase and the concentration of histamine in cell culture medium. **(B)** The effect of VIP and (or) aVIP on the activity of β-hexosaminidase, tryptase and the concentration of histamine in the cell culture medium. **(C)** The effect of *L. casei* ATCC 393 and (or) VIP on the activity of β-hexosaminidase, tryptase and the concentration of histamine in the cell culture medium. All data are presented as the mean ± S.E.M. (n=6). ^*^ *P* < 0.05, ^**^ *P* < 0.01, ^***^ *P* < 0.001. VIP means the PMMCs were exposed to VIP. *L* means the PMMCs were exposed to *L. casei* ATCC 393. aVIP means the PMMCs were exposed to aVIP. aVIP-VIP means the PMMCs were exposed to VIP and aVIP. *L*-VIP means the PMMCs were exposed to *L. casei* ATCC 393 and VIP.

As shown in **Figure 1A**, compared with the normal control group, exposure to *L. casei* ATCC 393 or VIP significantly induced the activation of PMMCs, and promoted the release of MCs-related mediators including β-hexosaminidase, tryptase and histamine. However, aVIP had no effect on PMMCs.

### Effect of Interaction Between VIP and aVIP on PMMCs Activation

As shown in **Figure 1B**, compared with the normal control group, treatment with VIP significantly increased the concentration of β-hexosaminidase, tryptase and histamine in the supernatant of PMMCs. Exposure to aVIP alone had no effect on PMMCs. However, administration of aVIP significantly inhibited VIP-induced activation of PMMCs.

### Effect of VIP Mediated *L. casei* ATCC 393 on Activation of PMMCs

As shown in **Figure 1C**, compared with the normal control group, both VIP and *L. casei* ATCC 393 treatments increased the β-hexosaminidase, tryptase and histamine concentration in the supernatant of PMMCs. However, *L. casei* ATCC 393 pretreatment significantly inhibited the VIP-induced activation of PMMCs and the release of MCs-related mediators.

### Effects of VIP and aVIP on ETEC K88- and LPS-Induced Activation of PMMCs

**Figure 2:**
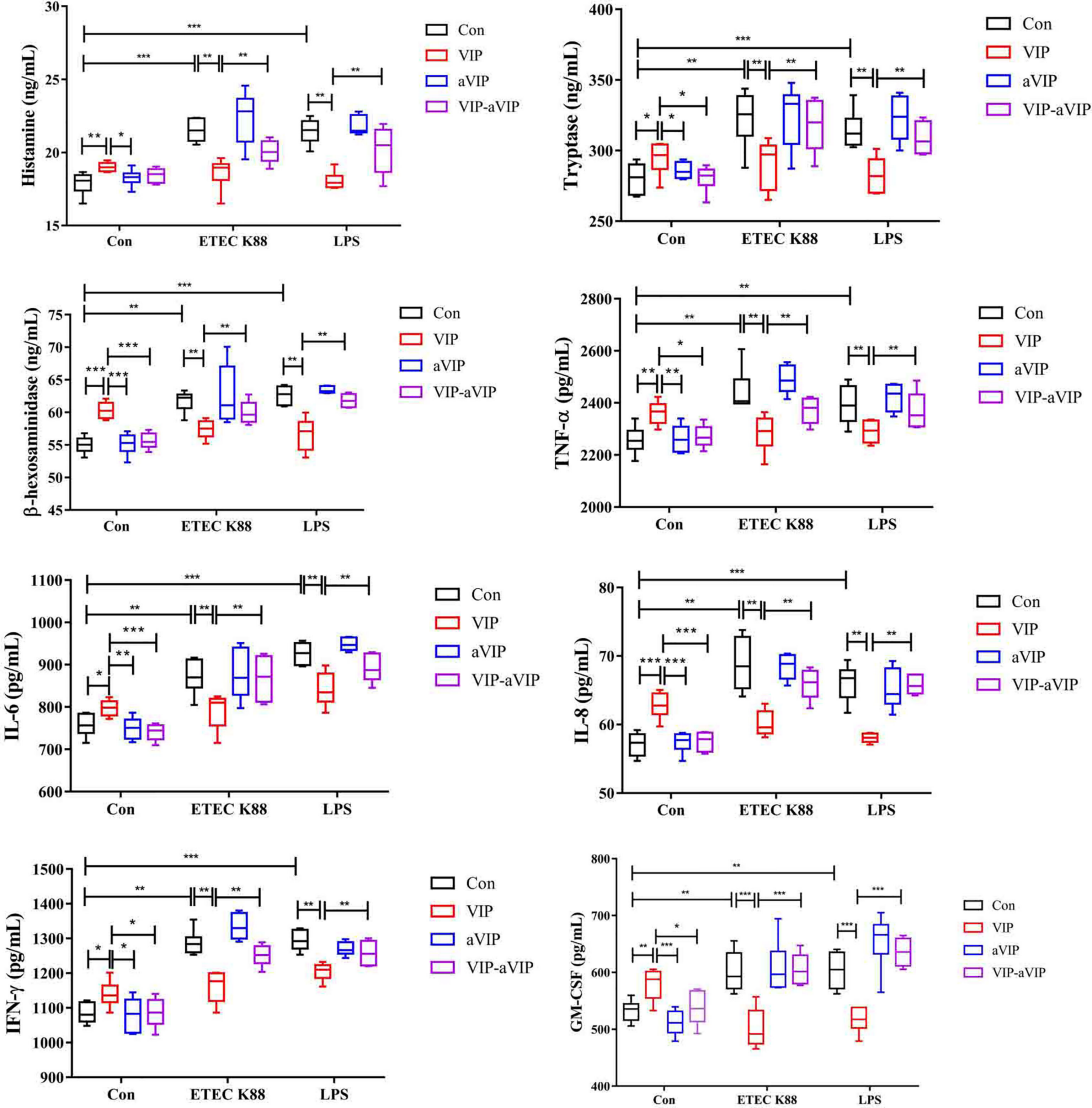
Effect of the interaction between vasoactive intestinal peptide (VIP) and VIP receptor antagonist (aVIP) on the degranulation of porcine mucosal mast cells (PMMCs) induced by Enterotoxigenic *Escherichia coli* K88 (ETEC K88) and lipopolysaccharide (LPS). The cells were treated with the supernatants of 1×10^8^ CFU/mL of ETEC K88 culture medium and 0.1μM LPS for 2h. The cells were treated with 0.1μM VIP and aVIP for 12h. All data are presented as the mean ± S.E.M. (n=6). ^*^ *P* < 0.05, ^**^ *P* < 0.01, ^***^ *P* < 0.001. VIP means the PMMCs were exposed to VIP. aVIP means the PMMCs were exposed to aVIP. aVIP-VIP means the PMMCs were exposed to VIP and aVIP.

As shown in **Figure 2**, compared with the normal control group, both ETEC K88 and LPS induced a significant increase of the TNF-α, IFN-γ, IL-6, IL-8, GM-CSF, β-hexosaminidase, tryptase and histamine levels in PMMCs supernatant. VIP significantly inhibited the increase of inflammatory factors, histamine, β-hexosaminidase and tryptase concentration in the supernatant of ETEC K88- and LPS-induced PMMCs. Administration with aVIP alone had no effect on ETEC K88- and LPS-induced activation of PMMCs. However, aVIP pretreatment significantly inhibited the regulatory effect of VIP on ETEC K88- and LPS-induced activation of PMMCs.

### Effects of aVIP and/or *L.casei* ATCC 393 on the Body Weight of Mice

**Figure 3:**
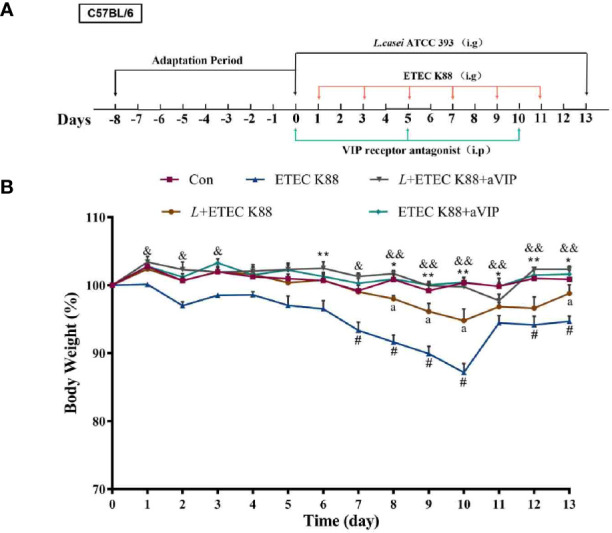
The experimental scheme and the change of body weight during the entire experimental period. The concentrations of both *L*. *casei* ATCC 393 and ETEC K88 were 1×10^8^ CFU/mL, and the drug concentration of aVIP was 10nmol/kg BW. **(A)** The experimental scheme. **(B)** The change of body weight. All data were presented as mean ± S.E.M. (n=10). *P < 0.05, **P < 0.01; ^#^ *P* < 0.05; ^&^ *P* < 0.05, ^&&^ *P* < 0.01; ^a^ *P* < 0.05. * means ETEC K88 infected group *v.s*. Con; # means *L* + aVIP +ETEC K88 *v.s*. ETEC K88 infective model group; & means aVIP + ETEC K88 *v.s*. ETEC K88 infected group; a means *L*+ETEC K88 v.s. ETEC K88 infected group. aVIP means the mice administered with the VIP receptor antagonist by i.p. injection. *L* means the mice orally received *L*. *casei* ATCC 393. ETEC K88 means the mice orally received ETEC K88.

The change of body weight of the mice during the whole experiment is shown in **Figure 3B**. According to the overall trend, except for the ETEC K88-infected group and *L. casei* ATCC 393 protection group, the body weight of the other groups remained relatively stable. On the days 6, from days 8 to 13, the body weight of mice in the ETEC K88-infected group was significantly lower than that in the normal control group. The groups that were orally administered *L. casei* ATCC 393, aVIP and *L. casei* ATCC 393 + aVIP all showed a significant alleviation of the reduction of body weight caused by ETEC K88. Moreover, the body weight of mice treated with *L. casei* ATCC 393 + ETEC K88+aVIP were significantly higher than that of mice in the *L. casei* ATCC 393 + ETEC K88 co-treated group.

### Effects of aVIP and/or *L. casei* ATCC 393 on Intestinal Morphology and Intestinal Barrier Function in Mice Challenged by ETEC K88

**Figure 4:**
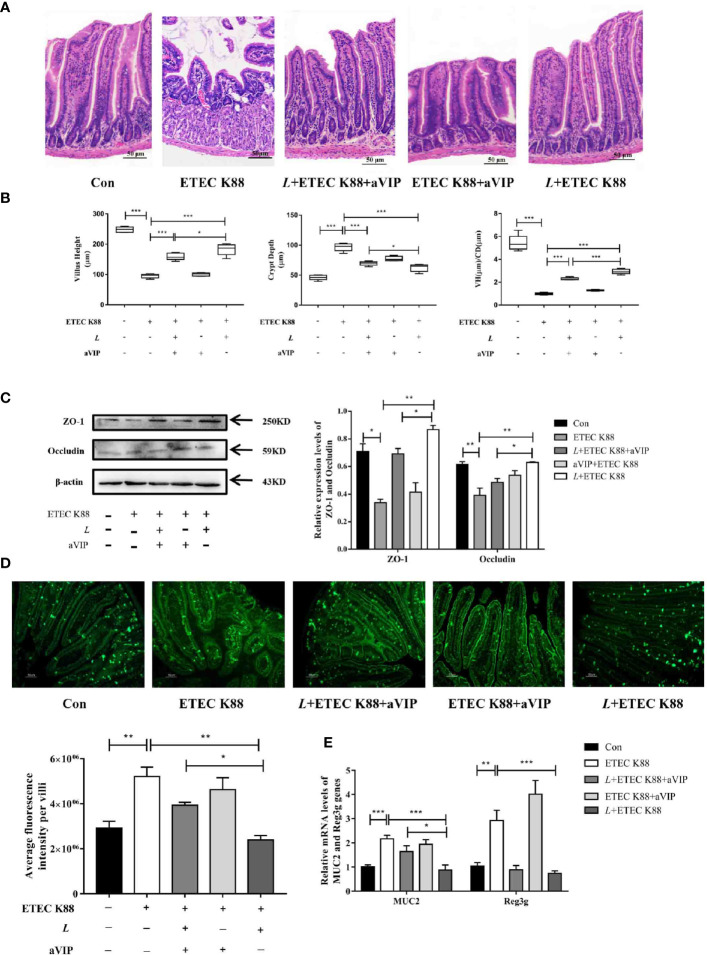
Effects of VIP receptor antagonist (aVIP) and *L. casei* ATCC 393 on intestinal barrier function. **(A)** The histomorphology of proximal duodenum was observed by hematoxylin-eosin (H&E) staining. **(B)** Quantitative analysis of villus height and crypt depth. **(C)** Expression levels of tight junction proteins ZO-1 and Occludin. **(D)** Immunofluorescence of MUC2 protein in duodenum. **(E)** The expression levels of MUC2 and Reg3g genes. All data are presented as the mean ± S.E.M. (n=4). ^*^ *P* < 0.05, ^**^ *P* < 0.01, ^***^ *P* < 0.001. aVIP means the mice were intraperitoneally (i.p.) injected with aVIP. *L* means the mice orally received *L. casei* ATCC 393. ETEC K88 means the mice orally received ETEC K88.

As shown in **Figures 4A, B**, compared with the normal control group, infection by ETEC K88 caused a significant increase in crypt depth (CD), a significant decrease in villus height (VH) and VH/CD of duodenum. Orally administered *L. casei* ATCC 393 significantly alleviated the above phenomenon. However, compared with the ETEC K88-infected group, the administration of aVIP alone had no effect on the VH, CD and VH/CD in mice exposed to ETEC K88. In addition, administration of aVIP significantly inhibited the effects of *L. casei* ATCC 393 on the VH, CD and VH/CD. As shown in **Figure 4C**, ETEC K88 significantly reduced the expression levels of ZO-1 and Occludin. *L. casei* ATCC 393 significantly alleviated the occurrence of the above phenomenon. aVIP showed antagonistic effect with *L. casei* ATCC 393 and inhibited the protective effect of *L. casei* ATCC 393 on intestinal barrier function. As shown in **Figure 4D**, compared with the control group, the expression levels of MUC2 protein in ETEC K88 group was significantly increased. Administration of *L. casei* ATCC 393 significantly inhibited the above phenomenon. However, aVIP had antagonistic effect on the regulatory effect of *L. casei* ATCC 393. As shown in **Figure 4E**, *L. casei* ATCC 393 significantly alleviated the increase of MUC2 and Reg3g mRNA expression levels induced by ETEC K88. However, the regulatory effects of *L. casei* ATCC 393 on MUC2 expression was inhibited by aVIP under ETEC K88 challenge.

### Effects of aVIP and/or *L. casei* ATCC 393 on Intestinal Mucosal Immunity in Mice Challenged by ETEC K88

**Figure 5:**
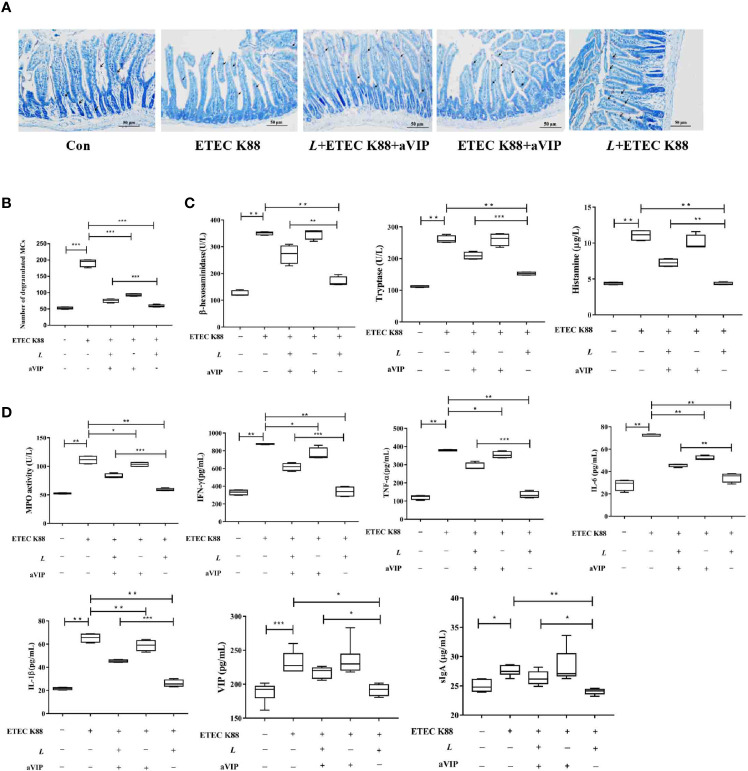
Effect of VIP receptor antagonist (aVIP) and *L. casei* ATCC 393 on the number of degranulated mast cells (MCs) in the ileum of mice challenged by ETEC K88 and the release of mast cells (MCs)-related mediators. **(A)** The number of degranulated MCs in proximal ileum was observed by Toluidine Blue (TB) staining. **(B)** Quantitative analysis of the number of MCs. **(C)** Effects of VIP receptor antagonist (aVIP) and *L. casei* ATCC 393 on the release of mast cells (MCs)-related mediators. **(D)** Effects of VIP receptor antagonist (aVIP) and *L. casei* ATCC 393 on serum myeloperoxidase (MPO) activity, VIP, sIgA and cytokines levels in mice challenged by ETEC K88. All data are presented as the mean ± S.E.M. (n=4) ^*^ *P* < 0.05, ^**^ *P* < 0.01, ^***^ *P* < 0.001. aVIP means the mice were intraperitoneally (i.p.) injected with aVIP. *L* means the mice orally received *L. casei* ATCC 393. ETEC K88 means the mice orally received ETEC K88.

TB staining of MCs in the proximal ileum of mice reveals, as shown in **Figures 5A, B**, that the number of total degranulated MCs in the proximal ileum of ETEC K88-infected mice was significantly higher than that in the normal control mice. Also, *L. casei* ATCC 393 or (and) aVIP treatment significantly attenuated the ETEC K88-induced increase in the number of total degranulated MCs in the proximal ileum. However, compared with the *L. casei* ATCC 393 treatment group, i.p. administration of aVIP significantly inhibited the regulatory effect of *L. casei* ATCC 393 on ileal mucosal MCs in mice infected by ETEC K88. As shown in **Figures 5C, D**, compared with the normal control group, infection by ETEC K88 significantly increased serum TNF-α, IL-6, IL-1β, IFN-γ, VIP, sIgA and histamine levels, as well as the β-hexosaminidase, tryptase and MPO activities. However, pretreatment with *L. casei* ATCC 393 or (and) aVIP significantly inhibited the increase of TNF-α, IL-6, IL-1β, IFN-γ levels, as well as the MPO activities induced by ETEC K88. *L. casei* ATCC 393 significantly inhibited the increase of sIgA and VIP levels induced by ETEC K88. Moreover, aVIP exhibited significantly antagonistic effect on the regulation of the release of TNF-α, IL-6, IL-1β, IFN-γ, VIP, sIgA and MCs-related mediators by *L. casei* ATCC 393.

### Effect of aVIP and/or *L. casei* ATCC 393 on The Microbial Community in Cecum of Mice Challenged by ETEC K88

**Figure 6:**
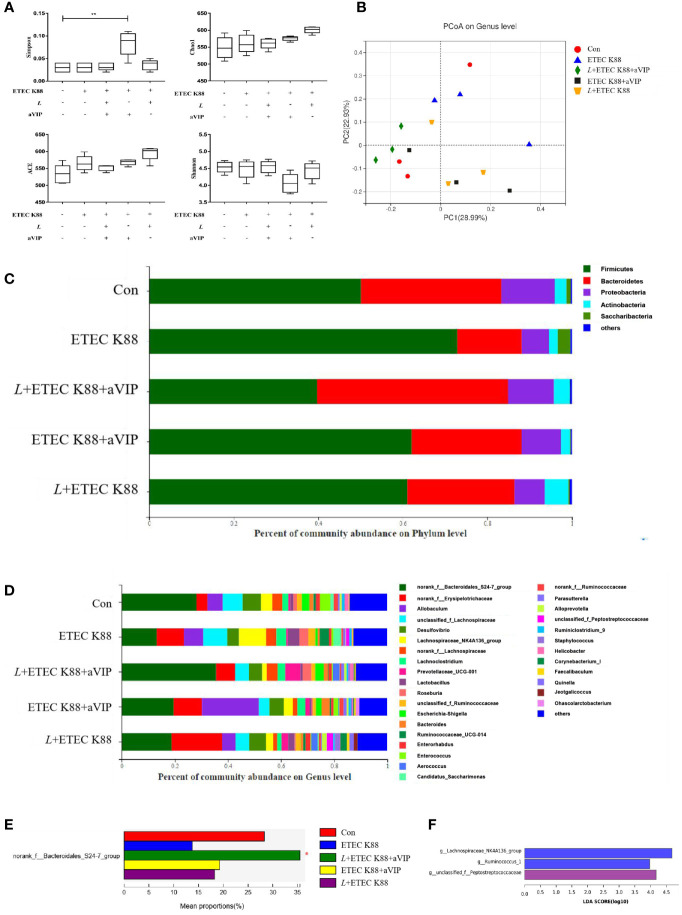
Effect of *L. casei* ATCC 393 and VIP receptor antagonist (aVIP) on microbial community in cecum of mice challenged by ETEC K88. **(A)** α-diversity analysis. **(B)** PCA plot of microbial community composition showed the compositional variance. **(C)** Differences in composition of microbial communities at phylum level. **(D)** Differences in composition of microbial communities at genus level. **(E)** LEfSe analysis of microbiota. All data are presented as the mean ± S.E.M. (n=4). ^*^ *P* < 0.05, ^**^ *P* < 0.01. aVIP means the mice were intraperitoneally (i.p.) injected with aVIP. *L* means the mice orally received *L. casei* ATCC 393. ETEC K88 means the mice orally received ETEC K88.

As shown in **Figure 6A**, compared with the other groups, administration of aVIP significantly increased the α-diversity of cecum microbiome. As for the ACE, Chao1 and Shannon index, there was no significant difference between each experimental group. As shown in **Figure 6B**, there was no significant difference for β-diversity in the genus level among each experimental group. In **Figure 6C**, there was no significant difference among those groups in the phylum level. However, as shown in **Figures 6D, E**, compared with the control, ETEC K88 significantly reduced the abundance of *norank_f_Bacteroidales_S24-7_group.* However, *L. casei* ATCC 393 and aVIP interventions alleviated the reduce of *norank_f_Bacteroidales_S24-7_group*. As shown in **Figure 6F**, LEfSe analysis showed that *g_Lachnospiraceae_NK4A136_group* and *g_Ruminococcus_1* were dominant species in mice challenged by ETEC K88, and *g_unclassified_f_Peptostreptococcaceae* and *f_Peptostreptococcaceae* were dominant species in mice administered with *L*+ETEC K88.

### Effect of aVIP and/or *L. casei* ATCC 393 on the Mechanism of Intestinal Barrier Function

**Figure 7:**
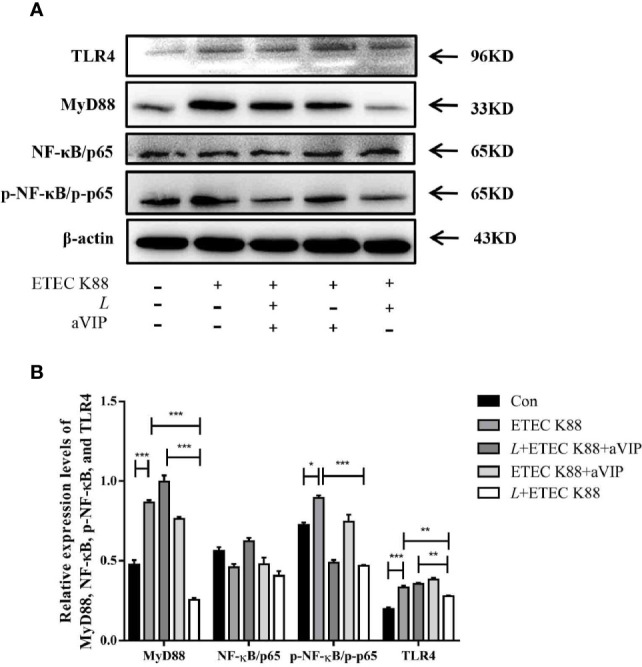
Expression levels of relevant inflammatory markers. **(A)** The expression levels of related inflammatory markers were detected by Western Blot. **(B)** Quantitative statistical results. All data were presented as mean ± S.E.M. (n=3). *P < 0.05, **P < 0.01, ***P < 0.001.

As shown in **Figures 7A, B**, compared with the control group, ETEC K88 challenge improved the expression levels of MyD88, p-NF-κB and TLR4. However, administration of *L. casei* ATCC 393 significantly inhibited the upregulation of MyD88, p-NF-κB and TLR4 induced by ETEC K88. Moreover, aVIP intervention exhibited antagonistic effects on *L. casei* ATCC 393.

## Discussion

The intestinal mucosal immune barrier plays a critical role in maintaining host homeostasis (24, 25). Studies have shown that intestinal barrier dysfunction is closely associated with the occurrence and development of various diseases, such as inflammatory bowel disease (IBD), chronic kidney disease, type II diabetes, fatty liver and heart disease (26, 27). Intestinal symbiotic bacteria and exogenous probiotics work together to maintain the integrity of the intestinal barrier, meaning that probiotics protect the intestinal barrier function (28). *Lactobacilli* MTCC 5690, LrhS3, Lp9, Lp4 and Lr120 can improve intestinal barrier function through toll-like receptor 2 (TLR2)- and toll-like receptor 4 (TLR4)-mediated mechanism and regulation of the expression of mucin 2 (MUC2) and tight junction proteins (29). The mixed probiotics of *bifidobacteria*, *Lactobacillus acidophilus* and *Enterococcus faecalis* can reduce the dextran sodium sulfate salt (DSS)-induced intestinal inflammation, improve multiple barrier functions, increase mucosal integrity, enhance transepithelial electrical resistance, reduce the permeability of the epithelium and endothelium to macromolecules, and increase the abundance of *bifidobacteria*, *lactobacillus* and *bacteroides* (30). In this study, we found that oral administration of *L. casei* ATCC 393 effectively protected the intestinal histomorphology and intestinal barrier function, and improve the ETEC K88-induced cecum microbiome dysbiosis.

MCs are fundamental elements of the intestinal barrier (18), MCs as important immunological effector cells play a key regulatory role in adaptive and innate immunity (31). In pathological conditions, MCs release pro-inflammatory compounds, including cytokines (32). Our preliminary research indicated that *L. casei* ATCC 393 can relieve ETEC K88-induce intestinal barrier dysfunction *via* the TLRs/MCs pathway (14). However, the regulatory mechanism of the intestinal barrier function mediated by MCs has not been elucidated. According to the recent studies, it may be associated with the release of neurotransmitters (such as VIP, substance P), and inflammatory mediators (such as cytokines) (33, 34). MCs not only senses the stimulation of harmful substances through the numerous receptors on their surface, including Fc receptor, complement receptor, TLRs, neuropeptide receptors, such as VPACs and antimicrobial peptide receptor, but also can synthesize and release transmitters to act on mucosal epithelium, nerve and other immune cells (35). As an extremely important neuroendocrine immunomodulatory peptide, VIP may be strongly associated with the regulation of intestinal barrier function (19). VIP can have a good therapeutic effect on necrotizing enterocolitis by reducing the inflammatory response and the destruction of TJ proteins (36). Furthermore, DSS induced colitis was associated with VIP and VPAC1 receptors (37). Moreover, VIP regulates a variety of immune cells, including MCs (38). VIP protects testis from torsion injury by inhibiting the activation of MCs (39). Additionally, VIP can also play a protective role on septic mice by regulating the activation of MCs (40). In this study, we found that VIP promoted the activation of PMMCs, and inhibited the activation of PMMCs exposed to ETEC K88 or LPS. These results suggested that the regulatory effect of VIP on PMMCs activation was strongly associated with the physiological and pathological conditions of cells. However, *L. casei* ATCC 393 or the aVIP can inhibit the VIP-induced activation of PMMCs.

MCs are important mediators of allergic responses on host surfaces including the intestine. When MCs is challenged by an external stimulus, it may respond by degranulation. In this process, a number of powerful preformed inflammatory “mediators” are released, including cytokines, histamine, serglycin proteoglycans, and several MC-specific proteases: chymases, tryptases, and carboxypeptidase A (41). In this study, the high numbers of degranulated MCs were observed in mice exposed to ETEC K88. MCs not only senses the stimulation of harmful substances through many receptors on the surface (including Fc receptors, complement receptors, TLRs, neuropeptide receptors, antimicrobial peptide receptors, etc.), but also can synthesize and release transmitters to act on mucosal epithelium, nerve, and other immune cells (35). Intestinal flora not only produces ligands for pattern recognition receptors (PRRs) (42), but also releases neurotransmitters and neuromodulators that target specific nervous systems on the brain-gut axis (43). If a TLR on MCs is activated, myeloid differentiation factor 88 (MyD88) and MyD88-adaptor like (MAL)/Toll-interleukin 1 receptor domain containing adaptor protein (TIRAP) associate and promote nuclear factor kappa-B (NF-κB) translocation to the nucleus resulting in cytokines transcription (44). TLR4 can be activated by LPS from Gram-negative bacteria (45). Antimicrobial peptides themselves have the function of protecting epithelial barrier and adjusting intestinal microbial balance (46). Probiotic *L. rbamnosus* Lc705 and *L. rbamnosus* GG could diminish mast cell activation (47). In this study, we observed that orally given *L. casei* ATCC 393 significantly inhibited the activation of ileal mucosal MCs in mice infected by ETEC K88. However, administration of aVIP by intraperitoneal injection abolished the regulatory effect of *L. casei* ATCC 393 on MCs activation in ileum, reduced the serum VIP level, and alleviated the inflammatory response in ETEC K88-infected mice. aVIP (VIP_6-28_) is the VIP receptor antagonist, which can competitively bound to the VPACs on the surface of MCs cells, counteracting the activation of endogenous VIP on MCs. In addition, administration of *L. casei* ATCC 393 or aVIP significantly alleviated the intestinal microbiome dysbiosis. Moreover, administration of *L. casei* ATCC 393 inhibited the activation of TLR4/MyD88/NF-κB signaling pathway induced by ETEC K88. VIP is an important secretomotor transmitter extensively expressed throughout the intestinal mucosa. Stress increased epithelial permeability, an effect that was largely blocked by a VIP receptor antagonist, and also the mast cell stabilizer doxantrazole (21). The above research results support the functional observations in the current study. The role of VIP in regulating anti-inflammatory- proinflammatory balance and intestinal barrier function has been demonstrated in humans and mice (20, 48, 49). The regulation of VIP on MC function may be bidirectional. It has been reported that VIP can affect the secretory activity of MCs, but the results are contradictory (38, 50). Therefore, the roles of VIP and mast cells in the regulatory effect of probiotics on intestinal barrier function require confirmation in further experiments.

## Conclusions

The regulatory effect of VIP on MCs activation may be related to the physiological and pathological conditions of cells. *L. casei* ATCC 393 inhibited the ETEC K88- and LPS-induced activation of intestinal mucosa MCs, and alleviated the intestinal mucosal injury in mice challenged by ETEC K88. VIP receptor antagonist abolished the protective effect of *L. casei* ATCC 393 on barrier function. Therefore, we speculated that the mechanism of *L. casei* ATCC 393 regulating intestinal mucosal injury may be related to VIP/MCs-mediated signaling pathway. However, the roles of VIP/MCs in the regulatory effects of probiotics on intestinal mucosal immune barrier function need further confirmation. This study is useful because it revealed the interaction of probiotics with enteric neuro-immunity and intestinal barrier function. *L. casei* ATCC 393 may be investigated as a potential and promising microecological food or feed additives in order to modulate intestinal barrier dysfunction.

## Data Availability Statement

The original contributions presented in the study are included in the article/supplementary material. Further inquiries can be directed to the corresponding authors.

## Ethics Statement

The animal study was reviewed and approved by Laboratory Animal Welfare and Ethics Committee of Northwestern Polytechnical University.

## Author Contributions

XS: the design of this study, supervising of this project. SP: implementation of *in vivo* experiments, analysis and interpretation of data. YMG and FZ: critical revision of the article for important intellectual content. SY: implementation of in vitro experiments, analysis and interpretation of data. YC: critical revision of the article for important intellectual content. LQ: critical revision of the article for important intellectual content. XD: critical revision of the article for important intellectual content. All authors contributed to the article and approved the submitted version.

## Funding

This work was supported by the National Natural Science Foundation of China (No.31672435 and No.32072746), the Key Research and Development Program of Shaanxi Province (No.2021NY-004), and Undergraduate Training Programs for Innovation and Entrepreneurship (No. S202010699424 and No. S202010699031X).

## Conflict of Interest

The authors declare that the research was conducted in the absence of any commercial or financial relationships that could be construed as a potential conflict of interest.

## Publisher’s Note

All claims expressed in this article are solely those of the authors and do not necessarily represent those of their affiliated organizations, or those of the publisher, the editors and the reviewers. Any product that may be evaluated in this article, or claim that may be made by its manufacturer, is not guaranteed or endorsed by the publisher.
